# Analysis of hand environment factors contributing to the hand surface infection barrier imparted by lactic acid

**DOI:** 10.1111/srt.13078

**Published:** 2021-09-16

**Authors:** Kaori Hayashi, Ichiro Mori, Kouske Takeda, Yasuhiro Okada, Atsuko Hayase, Takuya Mori, Yuki Nishioka, Kenji Manabe

**Affiliations:** ^1^ Personal Health Care Products Research Laboratories Kao Corporation Tokyo Japan; ^2^ Analytical Science Laboratories Kao Corporation Wakayama Japan; ^3^ Biological Science Laboratories Kao Corporation Tochigi Japan

**Keywords:** antimicrobial activity, contact infection, hand surface infection barrier, lactic acid, skin pH, skin temperature

## Abstract

**Background:**

Organic acids on the surface of human hands contribute to the barrier against transient pathogens. This is the first study to explore the synergistic contribution of lactic acid and other hand environment‐related features on the antibacterial properties of the hand surface.

**Materials and Methods:**

We estimated the contribution of fingerprint depth, skin pH, stratum corneum water content, skin temperature, and sweat rate of the hands to the infection barrier using an observational survey of 105 subjects. The relationship between each factor and the antibacterial activity of the hands was analyzed using Pearson's correlation coefficient. We performed molecular dynamics simulations to study the interaction between lactic acid and bacterial membranes.

**Results:**

The amount of lactic acid on the hands and skin temperature contributed positively to the antimicrobial activity (r = 0.437 and *P* = 3.18 × 10^−6^, r = 0.500 and *P* = 5.66 × 10^−8^, respectively), while the skin pH contributed negatively (r = −0.471, *P* = 3.99 × 10^−7^). The predicted value of the combined antimicrobial effect of these parameters was [antimicrobial activity] = 0.21 × [lactic acid] − 0.25 × [skin pH] + 0.26 × [skin temperature] + 0.98. The coefficient of determination (R^2^) was 0.50.

**Conclusion:**

The increase in the amount of non‐ionic lactic acid due to lower pH and improvement in the fluidity of the cell membrane due to higher temperatures enable the efficient transport of lactic acid into cells and subsequent antimicrobial activity. The proposed mechanism could help to develop an effective hand infection barrier technology.

## 1 INTRODUCTION

Contact infection through the “hands” is an important route in the transmission of pathogens by direct physical contact with infected individuals or by indirect contact through contaminated surfaces.^1^, ^2^ A study involving the surfaces of both dominant and non‐dominant hands revealed that more than 150 distinct species‐level bacterial phylotypes reside on the average palmer surface of human hands.^3^ Some of these bacteria are not part of the natural microbial microflora of the skin, but are transmitted pathogens that can cause various infectious diseases including gastrointestinal and respiratory illnesses.^4^, ^5^ These transmittable pathogens on hand surfaces are challenged by factors such as skin dryness, antimicrobial host defense, and exposures to soaps, detergents and other antimicrobial compounds, and UV radiation. From a public health perspective, the preventive effect of hand washing on infectious diseases has been strongly demonstrated epidemiologically.^5^, ^6^, ^7^ In addition, the perceptions of hand washing are closely associated with health behavior.^8^ However, existing hand hygiene practices are unable to fully prevent contact infections due to both the high frequency of self‐inoculation^9^ and the high stability of pathogens in the environment.^10^ A recent study revealed the prevalence of the collective habit of frequent face touching, even in a pandemic situation such as COVID‐19.^11^ As the outcome of the existing hand hygiene practices focuses on active interventions to remove and inactivate the pathogens attached to the hands, it is considered as difficult to manage the risk of contact infection in the context of habitual and social behaviors in daily life. In addition, intensive hand washing regimens can be practical challenging, and the frequent use of alcohol‐based hand sanitizers and gloves can lead to various adverse skin conditions.^12^, ^13^ Therefore, we have been focusing on the natural ability of humans against pathogens. Previous studies have shown that the surface of human hands naturally has some levels of antimicrobial activity.^10^, ^14^, ^15^ In addition, we reported that this antibacterial activity of the hands is suggested to be associated with the susceptibility to infections.^16^ Lactic acid has prominent antimicrobial properties.^17^, ^18^ Furthermore, our yet to be published data that include a comprehensive analysis of the components on the hand surface shows that not only the amount of lactic acid is abundant on the surface of the hands, but it also has a high positive correlation with the antimicrobial activity of the hands and that applying different amounts of lactic acid on the skin could improve the hand surface infection barrier.^16^

Given these, it is expected that a new hand hygiene technology could be developed by leveraging the hands’ innate antimicrobial mechanism in the form of a leave‐on lactic acid‐based formulation. Recent studies have highlighted the importance of the surface structure in the stability of pathogens.^19^, ^20^ Therefore, we assumed that the antimicrobial effects of hand surface components can differ at the individual level and hypothesized that additional hand environmental factors such as fingerprint depth, skin pH, stratum corneum water content, skin temperature, and sweat rate could have synergistic effects for improving the hand surface infection barrier. In this study, we investigated which hand environment factors are involved in the antibacterial activity of lactic acid and elucidated the mechanism of the hand surface infection barrier with the aim of creating a hand barrier technology.

## 2 MATERIALS AND METHODS

### 2.1 Bacterial strains and culture medium

The *Escherichia coli* NBRC3301 strain (NBRC, National Institute of Technology and Evaluation Biological Resource Center) was used. As a pre‐culture, a single colony was grown on soybean casein digest (SCD) agar medium (Nihon Pharmaceutical. Co., Ltd) and then inoculated into 4 mL of Luria‐Bertani (LB) liquid medium (Nihon Pharmaceutical. Co., Ltd.), and cultured overnight at 37°C and 180 rpm. Next, 1% of the obtained culture solution was inoculated into LB liquid medium, cultured for 15 hour, washed twice with sterile water, and stored on ice.^21^

### 2.2 Quantitative measurement of the bacterial count using the bioluminescence ATP assay

The bioluminescence ATP assay was used to count the number of viable bacteria by measuring the luminescence level of the ATP‐luciferase reaction,^22^ since the presence of ATP can be considered as proof of the presence of a living organism.^23^ The ATP luminescence intensity of the suspension was evaluated using a luciferin‐luciferase ATP assay reagent kit (Lucifer HS Set, Kikkoman Biochemifa Co.,), according to the manufacturer's instructions. Fifty microliters of ATP scavenging reagent were added to 500 μL of the sample, and the mixture was allowed to react for 30 minutes. ATP extract solution (100 μL) was added to 100 μL of the reaction solution and mixed for 20 seconds using a vortex mixer. Immediately after the reaction, 100 μL of luminescent reagent was mixed, and the luminescence intensity was measured using a luminometer (Lumitester C‐110, Kikkoman Biochemifa Co.,).

### 2.3 Study design

To investigate the physiological factors of the hands that are involved in the antibacterial activity, we conducted an observational study.

#### 2.3.1 Volunteer recruitment

Based on the number of variables included in the multiple regression model in this study, we estimated that a sample size of 60 would be adequate.^24^ Finally, a total of 106 healthy subjects, including 57 men and 49 women, were recruited randomly. The test was conducted from July 27 to August 28, 2018. Participants of Japanese and mongoloid races were recruited from the Kao Corporation with the following inclusion criteria: (1) healthy female or male aged between 20 and 60 years and (2) provided written informed consent to participate in the complete test process. Subjects were excluded if (1) they had skin symptoms such as atopic dermatitis and rosacea‐like dermatitis; (2) they had allergic symptoms due to the use of external medicines, cosmetics, quasi‐drugs, etc, in the past; (3) they had an external wound at the observation site; (4) they were taking antibiotics or antifungal agents and had taken it within the past month from the test date; and (5) those who were pregnant or may be pregnant and those who were less than 6 months after delivering the baby.

#### 2.3.2 Ethical approval

The study protocol, including sample collection, was reviewed and approved by the Ethical Committee of the Kao Corporation with approval number as S181‐180613. Informed written consent was obtained from all participants after the procedures were explained with documentation. All experiments were conducted in accordance with the principles of the Declaration of Helsinki.

#### 2.3.3 Sample collection

Subjects performed standard hand washing with a commercially available test soap　(Biore U Rg, Kao Corporation) formulated with alkyl ether sulfate, alkyl ether carboxylic acid, and alkyl glucoside without antimicrobial compounds,^25^ rinsed with tap water for 30 seconds, and then washed using purified water for 10 seconds. To avoid contact with the evaluation site, they wore polyethylene gloves (0950; SHOWA GLOVE Co.) for 2 hour of acclimation (20°C, 40% humidity). The measurement areas of antibacterial activity and physiological properties are shown in Figure S1(a‐h).

#### 2.3.4 In vivo quantitative evaluation of the surface infection barrier on the hands

For the quantitative evaluation, 10 µL of cultured *E coli* solution (OD = 10) was applied to an area of 4 cm^2^ on the palm of the hand (Figure S1a) and was collected 1 minutes later using a swab (BD‐BBL culture swab EZ; Becton Dickinson) soaked in physiological saline. The samples were collected twice by swabbing in one tube and then incubated in 1 mL of lecithin and polysorbate 80 (LP) solution (FUJIFILM Wako Pure Chemical Co.). The collected sample was shaken at 1000 rpm for 5 minutes using a high‐speed shaker (cute mixer) (CM‐1000, Tokyo Rikaki Co., Ltd.) to obtain the sample solution for the bioluminescence ATP assay. The initial number of bacteria used for the evaluation was prepared by adding 10 μL of *E coli* solution (OD = 10) to 1 mL of LP solution. In addition, the area where the bacteria were not applied was swabbed using the same procedure (Figure S1b) and used as a negative control. The amount of residual number of applied bacteria on the hand was calculated by subtracting the number of negative controls from the number on areas on which the sample was applied. Then, the antibacterial activity of the hands was evaluated relative to the initial viable bacterial number.

#### 2.3.5 Physiological measurements on hand

The amount of lactic acid, fingerprint depth, pH, and water content of the stratum corneum, temperature, and sweat rate were measured as follows. Regarding the measurement of lactic acid, an acrylic cylinder with an inner diameter of 1.5 cm was placed on the hand, 250 μL of pure water was added, and the mixture was pipetted. The concentration of L‐lactic acid was measured using Lactate Pro 2 (Arkray Co., Ltd.) using the extracted solution.^26^ The measurements were performed twice, and the values were averaged. The fingerprint depth was obtained as an index related to the surface shape of the hand. To measure the fingerprint depth, a replica of the thumb was made using a dental silicone resin (GC Examix Fine, GC Corporation) for 5 minutes with the thumb pressed. The fingerprint depth of the replica was measured by 3D imaging using a microscope (Digital Microscope VHX‐5000, KEYENCE Corp.).^27^ The pH of the skin was measured using a multi‐skin measuring instrument (MDD4) and skin pH meter (pH 905; Courage +Khazaka Electric GmbH). The water content of the stratum corneum was measured using a multi‐skin measuring instrument, MDD4 and Corneometer CM825 (Courage +Khazaka Electric GmbH). The skin temperature was measured with a contact thermometer TM‐300 (AS ONE Corp.).^28^ The sweat rate was considered as an index related to the water evaporation rate near the hand surface. The sweat rate was measured using a micro sweat meter TPL series (Techno Science Co., Ltd.).^29^

#### 2.3.6 Statistical analysis

JMP14 (manufactured by SAS Institute) was used for the statistical analysis. Since the amount of lactate in one of the 106 subjects was below the lower limit of detection (0.5 mmol L^−1^), this subject was excluded from the analysis, with n = 105. To investigate the relationship between each factor and the antibacterial activity of the hands, Pearson's correlation coefficient was calculated. In the Pearson correlation coefficient test, the significance level was set at <1%. To investigate the contribution of each factor, a multiple regression model was calculated based on the least‐squares method with in vivo antibacterial activity as the objective value. The Bayesian information criterion was adopted to optimize the factors. To compare the contributions of each factor, the explanatory variables were scaled (maximum value = 1, minimum value = −1, average value = 0) in the model.

### 2.4 Evaluation of the bactericidal activity through the in vitro experiments

L‐Lactic acid is a natural enantiomer in humans and is present in human sweat in amounts higher than that of D‐Lactic acid.^30^, ^31^ In this study, we focused on the L‐form in the in vitro analysis. A lactic acid solution of 0.2 wt% was prepared using L‐lactic acid (Tokyo Chemical Industries Co., Ltd.). Regarding pH adjustment, 48% sodium hydroxide (Kanto Chemical Industries, Ltd.) and 0.1 M hydrochloric acid (Kanto Chemical Co., Inc) were used. To evaluate the bactericidal activity, 10 μL of *E coli* solution (OD = 10) was added to 190 μL of sample solution, mixed for 15 seconds, and then incubated for 30 minutes. After the reaction, neutralization was carried out by adding 10 μL of the reaction solution to 1 mL of the LP solution (Fujifilm Wako Pure Chemical Co.). As the initial number of bacteria used for the calculation, 10 μL of *E coli* solution (OD = 10) was added to 190 μL of physiological saline and further diluted 100‐fold with an LP diluted solution. The antibacterial activity of each sample was evaluated relative to the initial number of viable bacteria.

### 2.5 Molecular Dynamics (MD) simulation conditions

Bacterial membranes are one of the targets of antimicrobial agents. To enhance the efficacy of a lactic acid‐based technology in maintaining skin hygiene, we aimed to understand the interaction of lactic acid with the bacterial membrane. To this end, we investigated whether the increase in the antibacterial activity of lactic acid with temperature is associated with the permeability of lactic acid. The permeation coefficient (P) of L‐lactic acid was calculated by molecular dynamics (MD) simulation, which controls the distance between the centers of gravity in the membrane thickness direction (z direction) of one molecule of L‐lactic acid. The intermembrane model of *E coli* was composed of 74 molecules of 1,2‐dioleoyl‐sn‐glycero‐3‐phosphoethanolamine (DOPE), 20 molecules of 1,2‐dioleoyl‐sn‐glycero‐3‐phospho‐rac‐(1‐glycerol) (DOPE), and six molecules of tetraoleoyl cardiolipin (TOCL1), with reference to the report of Sharma et al^32^ Furthermore, 26 potassium ions were added for electrical neutralization, and 4553 water molecules were added to prepare a 2.25 nm thick water slab. For the calculation of the *P*‐value, the inhomogeneous solubility diffusion (ISD) model^33^ represented by Equation (1) was used.

(1) $$\frac{1}{P}=\int_{z1}^{z2}\frac{\exp[\beta \Delta G(z)]}{D(z)}dz,$$

*β* = 1/*k*_B_ *T*, *k*_B_ is the Boltzmann constant, ΔG is the solvation free energy of lactic acid, and D is the diffusion coefficient of lactic acid in the z direction. ΔG is the coordinate data between 200 ns of the lactic acid molecule constrained by the harmonic potential at the z position every 0.1 nm from the center of the membrane to the aqueous phase obtained by the umbrella sampling method,^34^ and D was obtained by applying Bayesian analysis.^35^ CHARMM36 was used for the force field parameters of phospholipids and lactic acid,^36^, ^37^ and TIP3P^38^ was used for the water model. A series of calculations was performed using Gromacs‐2018.^6^, ^39^

## 3 RESULTS

### 3.1 Statistical analysis for the contribution of physiological properties to the hand surface infection barriers

#### 3.1.1 Single correlation analysis for the estimation of factor contribution

**FIGURE 1:**
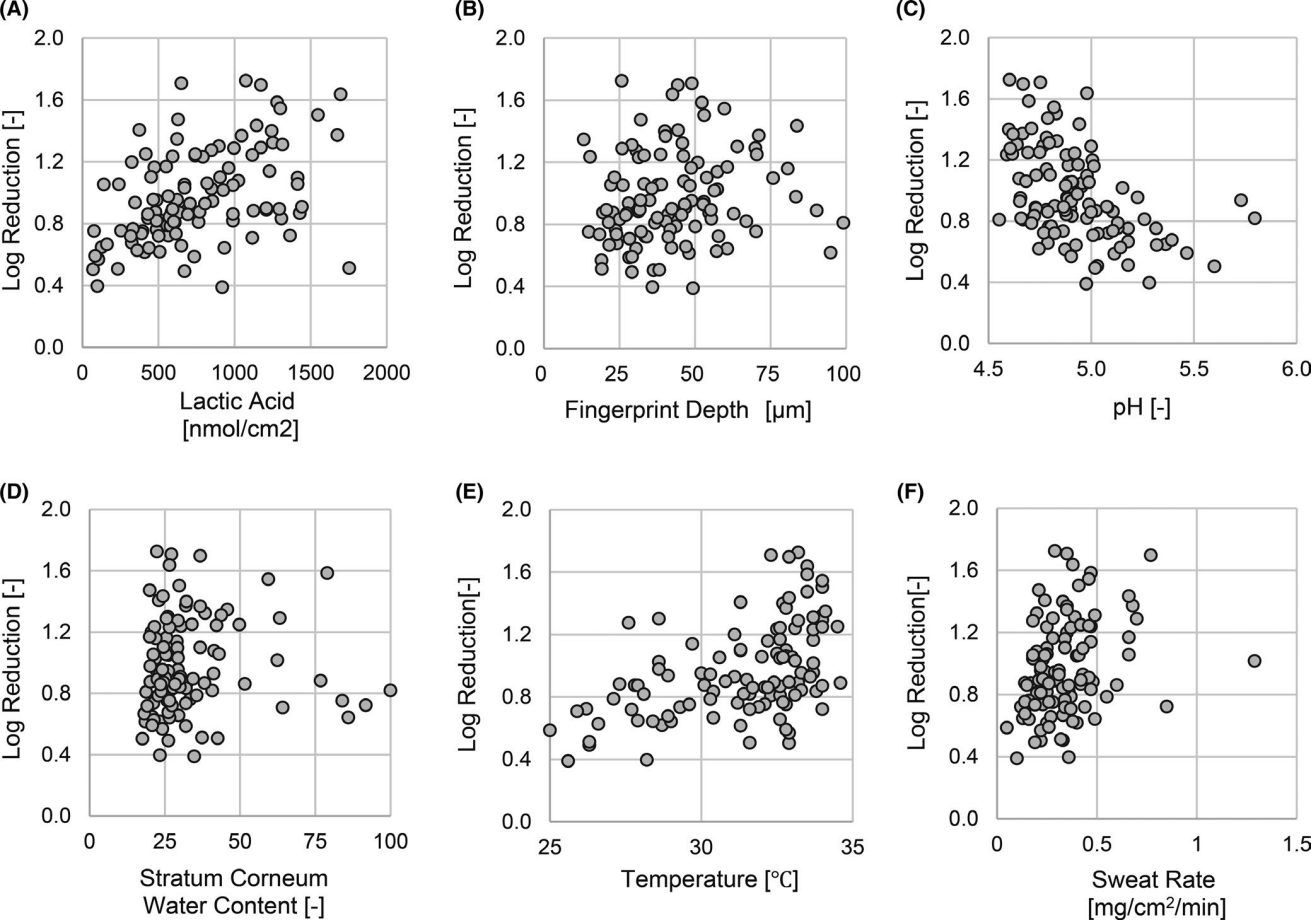
Contribution of lactic acid and physiological factors to the antibacterial activity of hands. The vertical axis represents the in vivo antibacterial activity of the hands, and the horizontal axis represents, (A) the amount of lactic acid on the hands, (B) fingerprint depth, (C) skin pH, (D) stratum corneum water content, (E) skin temperature, and (F) sweat rate. Antibacterial activity is shown as the log reduction value of *E coli*

To clarify the physiological properties involved in hand surface infection, a total of five factors (fingerprint depth, pH, stratum corneum water content, temperature, and sweat rate) related to hand properties and the amount of lactic acid on the hand surface were obtained from the subjects (n = 105), and the relationship between each factor and the antimicrobial activity of the hands was analyzed by simple correlation analysis. The log reduction value indicates the relative logarithmic reduction in viable bacteria 1 minute after the bacterial solution was applied to the hand. Figure 1 (A‐F) shows a scatter plot of the amount of each factor, including lactic acid, and the in vivo antimicrobial activity of the hand. As a result of testing the null hypothesis using Pearson's correlation coefficient for six factors, a significant correlation (*P*‐value < significance level *α* = 0.01) was found in the following four factors (Table 1). That is, the amount of lactic acid (r = 0.437, *P* = 3.18 × 10^−6^), skin pH (r = −0.471, *P* = 3.99 × 10^−7^), skin temperature (r = 0.500, *P* = 5.66 × 10^−8^), and sweat rate (r = 0.290, *P* = 2.68 × 10^−3^) were significantly correlated with antimicrobial activity.

**TABLE 1:** Correlation between each factor and in vivo antibacterial activity

Factor	Correlation Coefficient^1^	*P*‐value^2^
**Lactic acid [nmol/cm^2^]**	**0.437**	**3.18 × 10^−6^**
Fingerprint Depth [µm]	0.145	1.40 × 10^−1^
**pH [−]**	**‐0.471**	**3.99 × 10^−7^**
Stratum Corneum Water Content [−]	0.024	8.07 × 10^−1^
**Temperature [°C]**	**0.500**	**5.66 × 10^−8^**
**Sweat Rate [mg/cm^2^/min]**	**0.290**	**2.68 × 10^−3^**

Note

Correlation^1^ and *P*‐value^2^ were calculated using the Pearson correlation coefficients. Bold numbers indicate statistically significant differences (*P* < 0.01).

#### 3.1.2 Multiple regression analysis for the estimation of factor contribution

**FIGURE 2:**
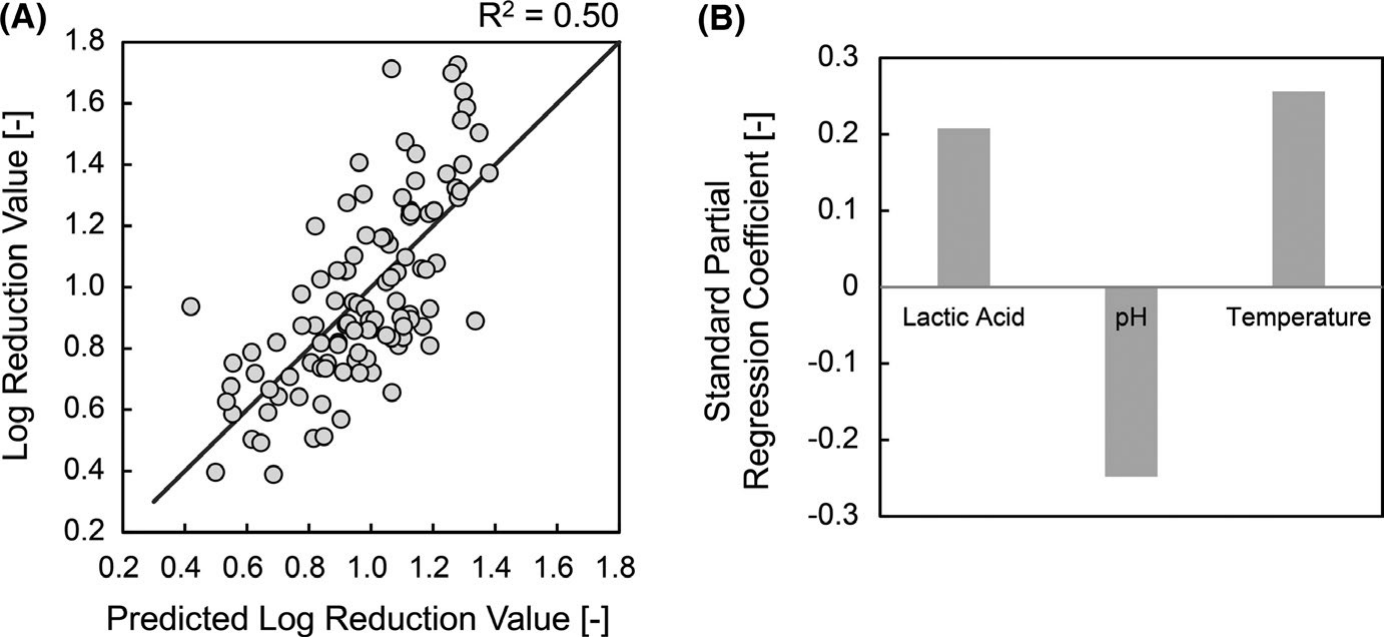
Multiple regression model of the in vivo antibacterial activity. A, Correlation between the measured and predicted values of the in vivo antibacterial activity of hands. The vertical axis represents the actual antibacterial activity, and the horizontal axis represents the antibacterial activity predicted by the multiple regression model. The predicted value [antimicrobial activity] = 0.21 × [lactic acid] − 0.25 × [skin pH] + 0.26 × [skin temperature] + 0.98. The coefficient of determination (R^2^) was 0.50. B, Standard partial regression coefficient of lactic acid, pH, and temperature

To investigate the contribution of each factor to the antimicrobial activity, a multiple regression model based on the least‐squares method with in vivo antimicrobial activity as the objective variable was created. By optimizing the model through variable selection using the minimum Bayesian information criterion (BIC) method, a multiple regression model with three factors, including lactic acid amount, skin pH, and skin temperature, was obtained. When these three factors were selected, the *P*‐values of the other factors, including the sweat rate, were not correlated (above 0.1) (Table 2). The multiple regression model formula was as follows: predicted value [antimicrobial activity] = 0.21 × [lactic acid] − 0.25 × [skin pH] + 0.26 × [skin temperature] + 0.98. The coefficient of determination (R^2^) was 0.50 (Figure 2A). Regarding the contribution of the three factors, the amount of lactic acid and skin temperature contributed positively, and the skin pH contributed negatively to the antimicrobial activity. The standard partial regression coefficients of the three factors were almost identical (Figure 2B).

**TABLE 2:** Parameters calculated using the multiple regression model

Factor	F‐value^1^	*P*‐value (Prob <F)^2^
**Lactic acid [nmol/cm^2^]**	**19.89**	**2.13 × 10^−5^**
Fingerprint depth [µm]	0.096	7.58 × 10^−1^
**pH [−]**	**17.63**	**5.78 × 10^−5^**
Stratum Corneum Water Content [−]	0.332	5.66 × 10^−1^
**Temperature [°C]**	**34.15**	**6.29 × 10^−8^**
Sweat Rate [mg/cm^2^/min]	1.702	1.95 × 10^−1^

Note

F‐value^1^ and *P*‐value^2^ were calculated using a multiple regression model based on the least‐squares method with in vivo antimicrobial activity as the objective variable. Bold numbers indicate statistically significant differences (*P* < 0.01).

Furthermore, in the analysis of the correlation between these factors, a significant negative correlation was found between the amount of lactic acid and skin pH (r = −0.258, *P* = 7.98 × 10^−3^) (Table 3). However, the correlation between the amount of lactate and skin pH was lower than that between each factor and the in vivo antimicrobial activity (r = 0.437, *P* = 3.18 × 10^−6^ and r = −0.471, *P* = 3.99 × 10^−7^, respectively) (Table 1). On the other hand, the sweat rate was significantly correlated with the amount of lactic acid (r = 0.397, *P* = 2.72 × 10^−5^) (Table 3), and this correlation was higher than that between the sweat rate and in vivo antimicrobial activity (r = 0.290, *P* = 2.68 × 10^−3^) (Table 1).

**TABLE 3:** Correlation between factors (correlation coefficient and *P*‐value)

	Lactic acid	pH	Temperature	Sweat rate
Lactic acid	1.00	‐	‐	‐
pH	**−0.258 (*P* = 7.98 × 10^−3^)**	1.00	‐	‐
Temperature	0.075 (*P* = 4.50 × 10^−1^)	−0.182 (*P* = 6.33 × 10^−2^)	1.00	‐
Sweat Rate	**0.397 (*P* = 2.72 × 10^−5^)**	−0.051 (*P* = 6.04 × 10^−1^)	0.147 (*P* = 1.33 × 10^−1^)	1.00

Note

Correlation coefficients and *P*‐values were calculated using the Pearson correlation coefficients. Bold numbers indicate statistically significant differences (*P* < 0.01).

### 3.2 Verification of the effects of pH and temperature on antibacterial activity

#### 3.2.1 The effect of pH on the antimicrobial action of lactic acid

**FIGURE 3:**
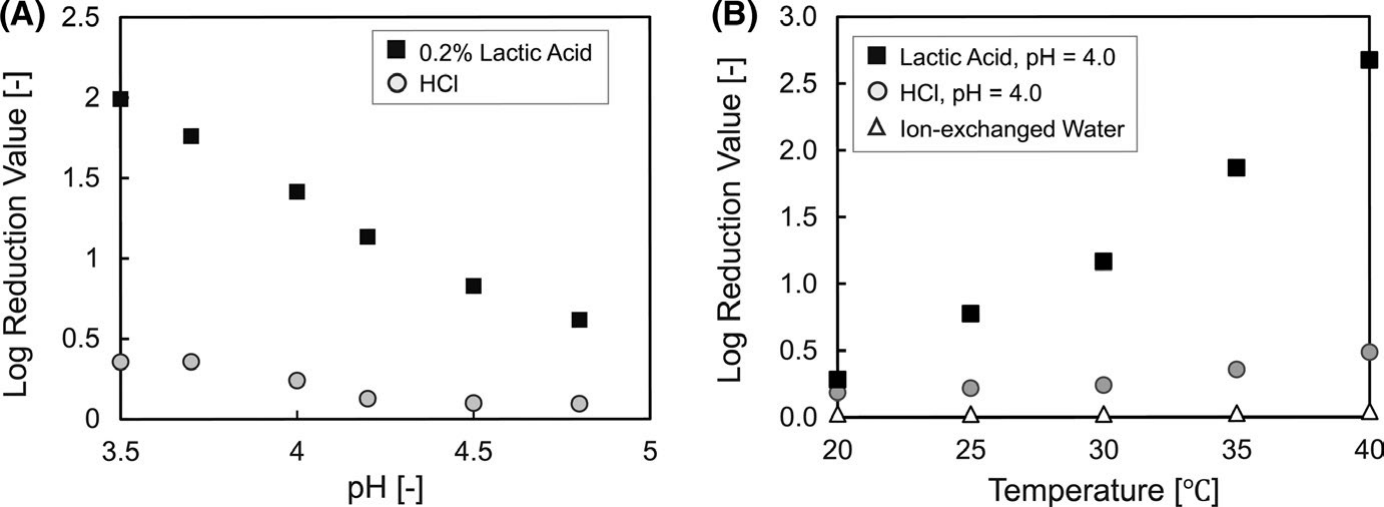
Verification of the effects of pH and temperature on antibacterial activity. A, pH dependence of antibacterial activity. The horizontal axis represents the pH of the sample, and the vertical axis represents the in vitro antibacterial activity of 0.2 wt% of L‐lactic acid under several pH conditions under 30°C. B, Temperature dependence of the antibacterial activity. The horizontal axis represents the temperature of the assay condition and the vertical axis represents the in vitro antibacterial activity of 0.2 wt% of L‐lactic acid (pH = 4.0), HCl (pH = 4.0), or ion‐exchanged water under several temperature conditions

Statistical analysis showed that there was a negative correlation between skin pH and in vivo antimicrobial activity, indicating that the lower the skin pH, the higher the in vivo antimicrobial activity. However, it is not clear whether this is due to the solitary effect of skin pH or the synergistic effect of the two factors, skin pH and lactic acid. Therefore, we compared the in vitro antimicrobial activities of the lactic acid solution with that of the HCl solution at the same pH (Figure 3A). In this experiment, antimicrobial activity was investigated at different pH values and the same concentration of L‐lactic acid (0.2 wt%). In the HCl solution, there was no correlation between pH and in vitro antimicrobial activity, and the value of the in vitro antimicrobial activity was quite low. On the other hand, in the lactic acid solution, the antimicrobial activity increased remarkably with decreasing pH.

#### 3.2.2 The effect of skin temperature on the antimicrobial action of lactic acid

Statistical analysis of the results from the clinical survey showed that *the* in vivo antimicrobial activity was positively correlated with skin temperature. Therefore, we investigated the temperature dependence of the in vitro antimicrobial activity using an L‐lactic acid solution (pH 4), HCl solution (pH 4), and ion‐exchanged water (Figure 3B). No definite correlation was found between the temperature and in vitro antimicrobial activity of HCl solution (pH 4) or ion‐exchanged water, and these values were low. In contrast, in the lactic acid solution (pH 4), the in vitro antimicrobial activity increased remarkably as the temperature increased.

### 3.3 Verification of the effect of temperature on the cell membrane penetration of lactic acid by MD simulations

**FIGURE 4:**
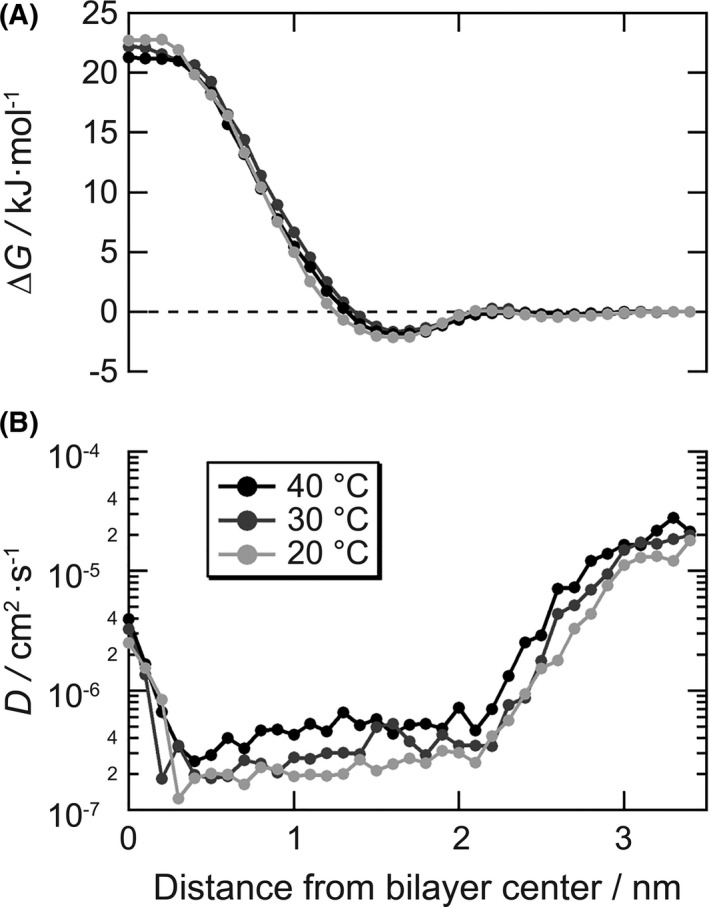
Physicochemical parameters that determine permeability of lactic acid in the intermembrane model of *E coli*. A, Solvation free energy of a lactic acid based on the water phase and B, diffusion coefficient of a lactic acid in the normal direction in each position

Furthermore, we investigated the effect of temperature on the penetration of lactic acid against the cell membrane using MD simulations. The solvation free energy ΔG in the permeation of lactic acid into the *E coli* intermembrane model showed almost the same profile under the conditions of 20, 30, and 40°C, and the values were a minimum of −2 kJ mol^−1^ near the glycerophosphate on the outside of the membrane, and approximately 20 kJ mol^−1^ at the center of the membrane, respectively (Figure 4A). The membrane thickness was roughly estimated to be approximately 3.4 nm, twice the distance from the center of the bilayer to the glycerophosphate at each temperature condition. On the other hand, the diffusion coefficient (D) of lactic acid in the z direction at 40°C was approximately twice that at 20°C and 1.5 times that at 30°C (Figure 4B). The lactate permeation coefficient (P) calculated based on the ISD model is 4.0 × 10^−4^ cm s^−1^ at 20°C, 6.0 × 10^−4^ cm s^−1^ at 30°C, and 1.6 × 10^−3^ cm s^−1^ at 40°C, indicating that the higher the temperature, the greater the membrane permeability of lactic acid.

## 4 DISCUSSION

Bacteria grow and thrive on human skin, and many of them have adapted to survive in various regions of the skin surface. Human hands are one of the most dynamic regions for complex microbial habitats because of their continuous and varied exposure to different environmental surfaces. In this study, we investigated the contribution of specific hand surface characteristics to the infection barrier by conducting an observational study. As an earlier study suggested, between the two lactic acid isomers, L‐lactic acid was significantly more effective than D‐lactic acid for killing *E coli*,^30^, ^31^ we conducted the in vitro experiments with L‐lactic acid. We elucidated the mechanism of the hand surface infection barrier using an in vitro antibacterial activity assay and MD simulations.

The simple correlation of the observational study indicated that four factors, including the amount of lactic acid on the hand palm, skin pH, skin temperature, and sweat rate, were significantly correlated with the antibacterial activity of the hand surface. Earlier reports revealed that weak acids, such as lactic acid, may diffuse into the cell in their neutral form, leading to acidification of the intracellular milieu and growth inhibition. In addition, as a general trait of all living cells, the cellular decisions are made by the dynamic control of pH.^40^ In our study, the multiple regression analysis showed that the three factors, amount of lactic acid, skin pH, and skin temperature independently contributed to in vivo antibacterial activity. The sweat rate had a stronger correlation with the amount of lactic acid than the in vivo antimicrobial activity. This might be due to the presence of lactic acid,^41^ which is the main contributing factor to the antimicrobial activity in sweat. The parameters of the regression equation suggested that (1) an increase in the amount of lactic acid, (2) a decrease in pH, and (3) an increase in temperature would contribute to the improvement of the antimicrobial activity of the hand surface. The contribution rates of these three factors were almost the same in terms of the magnitude of the respective standard partial regression coefficients (Figure 2B). Since the coefficient of determination (R^2^) value of the regression equation was 0.50, the antimicrobial activity could be predicted with a certainty of approximately 50% by measuring the amount of lactic acid, pH, and temperature. Regarding the residual 0.50, it is possible due to the unexamined parameters such as organic acids, fatty acids, proteins, and inorganic salts on the hands, which might contribute to the antimicrobial activity on hands.^16^ Although previous studies have highlighted the importance of environmental moisture or humidity for the survival rate of pathogens,^42^ we could not find any significant correlation between the water content of the stratum corneum water and antimicrobial activity.

The results of the in vitro antimicrobial activity experiment using lactic acid solution with several pH values (Figure 3A) revealed that the synergistic effect of low pH and high amount of lactic acid is important to achieve a high antimicrobial activity on the hands. In general, most organic acids are weak acids, and they have anionic and non‐ionic type in an equilibrium state depending on the pH of the solution.^21^, ^43^ Then, only the non‐ionic acids can permeate the cell membrane and contribute to the antimicrobial activity through membrane destruction,^44^, ^45^ inhibition of essential metabolic reactions,^46^ disturbance of pH homeostasis in cells, ^44^, ^47^ and the accumulation of toxic anions.^48^ Usually, because of their membrane, gram‐negative bacteria are typically less susceptible to weak acids.^49^ Lactic acid is a weak acid with pKa =3.86, suggesting that non‐ionic lactic acid under low pH conditions can inactivate bacteria through the same mechanism, leveraging its ability to permeabilize the membrane of the bacteria. In addition, the antimicrobial activity of lactic acid is reported to depend on the temperature.^50^ The temperature dependence of the in vitro antibacterial activity of lactic acid (Figure 3B) suggests that the improvement of the antibacterial activity is due to the synergistic effect of temperature and lactic acid. A previous report showed that temperature affects the fluidity of the phospholipid bilayer membrane of bacteria.^51^ Thus, it is considered that the fluidity of the bacterial cell membrane improves as the temperature increases and the inflow of lactic acid inside the bacterium increases. Furthermore, we analyzed the mechanism by which lactic acid inactivates bacteria under higher temperature conditions using MD simulations. Generally, the solute flux is proportional to the permeability of the solute and the concentration difference on both sides of the membrane. Therefore, under the same concentration of solutes, the permeability may mainly contribute to the improvement of the in vivo antimicrobial activity in the early stage of solution contact. Consistent with the in vivo hypothesis that the antibacterial activity increases with increased inflow of lactic acid at higher skin temperatures, the permeation coefficient of lactic acid increased with an increase in temperature. Permeation of lactic acid is the molecular crossing process in the hydrophobic region of the membrane center where the solvation free energy of a lactic acid molecule is the highest. The free energy profiles were almost the same at all temperature conditions, whereas the diffusion coefficients showed a larger value with increasing temperature. Therefore, the improvement in the permeation coefficient was mainly attributed to the diffusion coefficient. The molecular scale behavior corresponds to the increasing fluidity of the membrane, which is attributed to the thermal fluctuation of phospholipids. We analyzed the lateral diffusion coefficient from the mean square displacement of carbon atoms at the terminal of the acyl group of phospholipids, as shown in Figure S2, and confirmed that the fluidity of the lipid bilayer increased with increasing temperature. These findings are useful for understanding the mechanism of the bactericidal effect of lactic acid because there are few reports analyzing the penetration of organic acids through bacterial membranes using MD simulations.

**FIGURE 5:**
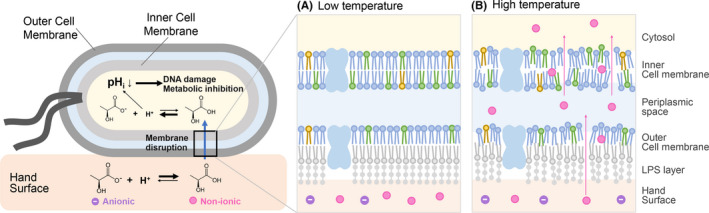
Proposed mechanism of the hand surface infection barrier. Here is the proposed mechanism of a strong hand surface infection barrier. Lactic acid can accumulate on the skin due to its high sweat rate, and low pH of the skin increases the non‐ionic nature of lactic acid, which can permeate the bacterial cell membrane, possibly due to diffusion. A, The permeability of lactic acid was low at low temperatures. B, High temperature of the skin improves the fluidity of the cell membrane by thermal fluctuation and promotes the permeation of lactic acid into the cell membrane, which leads to the inactivation of bacteria

This study is the first to clarify that the surface components and skin properties act synergistically on the hand to create high antibacterial activity. The in vitro antibacterial activity assay and MD simulations suggested that the mechanism of the strong hand surface infection barrier is as follows: (1) The lactic acid could be accumulated on the skin due to the high sweat rate. (2) Low pH of the skin could increase the non‐ionic nature of lactic acid, which can permeate the bacterial cell membrane, possibly due to diffusion. (3) The high temperature of the skin improves the fluidity of the cell membrane by thermal fluctuation and promotes the permeation of lactic acid into the cell membrane (Figure 5). These phenomena synergistically would contribute to the high antimicrobial activity on the hand. One limitation of our study is that it exclusively focused on the bactericidal effect of lactic acid against *E coli*. Future studies should not only explore the activity against other bacteria, but also against the viruses of the hand surface. In addition, to fully leverage the antimicrobial activity of lactic acid, it would be important in our future studies to assess other targets such as integrity of the cell membrane, biosynthesis of proteins and nucleic acids, and activity of critical enzymes and osmotic homeostasis, in addition to the membrane permeability and cytoplasm acidification.

## 5 CONCLUSION

The proposed mechanism in this study would lead to the construction of an effective leave‐on technology to improve the hand infection barrier. In addition, the statistical analysis provided a multiple regression model that predicted antibacterial activities based on the amount of lactic acid, pH, and temperature. Thus, this prediction might be used as a monitoring system for the hand surface infection barrier using a simple device without using bacteria. By combining leave‐on technologies and the monitoring system, we can provide a new hand hygiene model to improve the hand surface infection barrier at the appropriate time for individuals.

## CONFLICTS OF INTEREST

All authors are employees of the study sponsor, Kao Corporation, Tokyo, Japan.

## AUTHORS’ CONTRIBUTIONS

All authors have read and approved the manuscript. KH, YN, and KT wrote the manuscript. IM and AH conducted a clinical survey to analyze the relationship between physiological skin conditions and the antibacterial activity of the hand. KH conducted the statistical analysis of the clinical data. YO performed the in vitro antibacterial experiments. KT conducted MD simulations, and TM and KM supervised the study and reviewed the results.

## Supporting information

Fig S1

Fig S2
