# Supplementary figures and images for: Analysis of hand environment factors contributing to the hand surface infection barrier imparted by lactic acid
Source: Skin Res Technol. 2021 Sep 16;27(6):1135–44. doi: 10.1111/srt.13078 (PMC9293006; doi:10.1111/srt.13078)

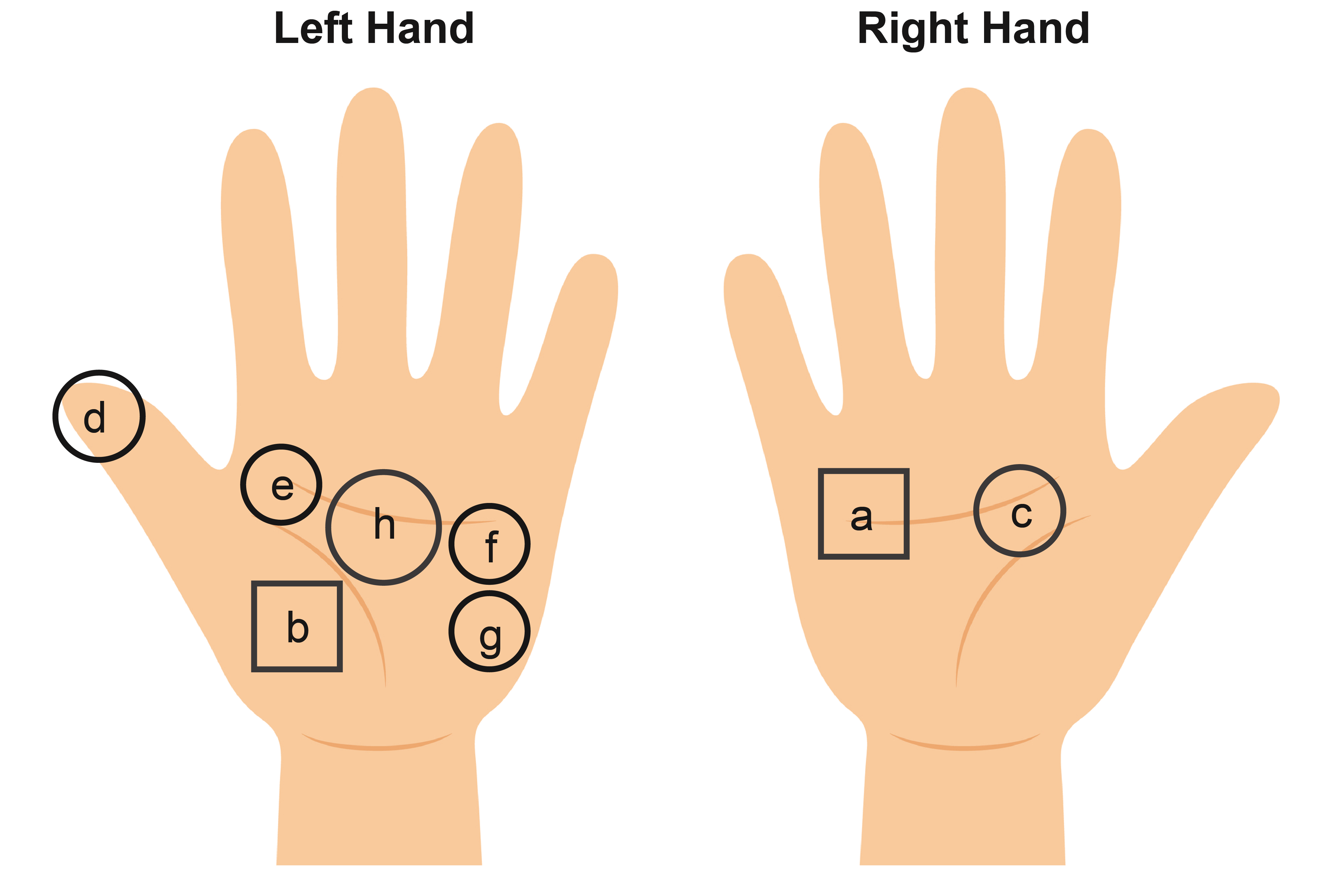

Supplement: Supplementary file 1 — Fig S1 [file SRT-27-1135-s002.tif]

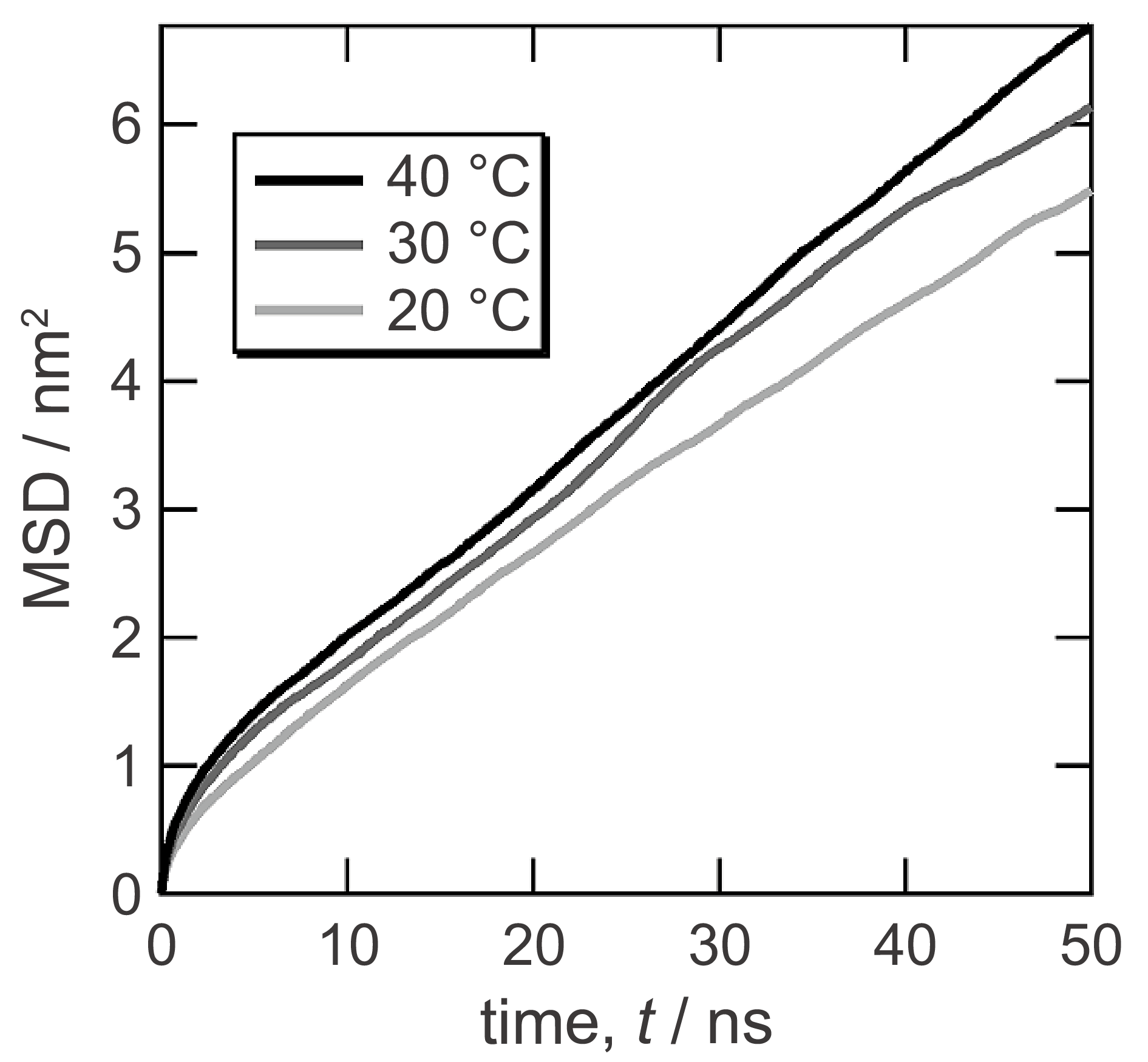

Supplement: Supplementary file 2 — Fig S2 [file SRT-27-1135-s001.tif]
